# Body Fat Moderates the Association of Olfactory Dysfunction with Dietary Intake in U.S. Middle-Aged and Older Adults: A Cross-Sectional Analysis of NHANES 2013–2014

**DOI:** 10.3390/nu14153178

**Published:** 2022-08-02

**Authors:** Surabhi Bhutani, Amanda C. McClain

**Affiliations:** School of Exercise and Nutritional Sciences, San Diego State University, San Diego, CA 92182, USA; amcclain@sdsu.edu

**Keywords:** olfactory function, hyposmia, anosmia, national health and nutrition examination survey, diet quality, healthy eating index, body fat, waist circumference

## Abstract

Background: Obesity relates to impaired olfactory function. Abnormal olfactory function is also associated with poor diet; however, whether obesity-related markers shape this relationship is unknown. Methods: Cross-sectional analysis (*n* = 1415, age > 40 years) of NHANES 2013–2014 examined body fat percent (BF%) and waist circumference (WC) as moderators of the relationship between olfactory function and diet. The olfactory function test identified adults with olfactory dysfunction (OD) or normal olfaction (NO). Validated 24 h recall captured nutrient intake and Healthy Eating Index-2010 scores. BF% and WC were measured. We tested adjusted linear regression models, with an interaction term between olfactory function and BF%/WC, for each nutrient or HEI score, and reported coefficients (β), standard errors (SE), and *p*-values for significant interaction terms. Results: In OD (9.5%; mean age 50.9 years, 95% CI 49.6, 52.2) compared with NO (mean age 49.3 years, 95% CI 48.8, 49.9), higher BF% was associated with higher intake of saturated fat (β (SE): 0.2 (0.1) g; *p* = 0.06) and percent of total calories from total fat (0.2 (0.1); *p* = 0.07), saturated (0.1 (0.004); *p* = 0.02), and monounsaturated fat (0.1 (0.1); *p* = 0.08); lower percent of total calories from carbohydrates (−0.2 (0.1); *p* = 0.09) and mg of sodium (−17.8 (09.6); *p* = 0.08); and a higher (healthier) refined grain score (0.1 (0.1); *p* = 0.04). Higher WC was associated with higher refined grain scores (0.01 (0.02); *p* = 0.01) in OD. Conclusion: BF% may shape dietary intake and quality in OD. Longitudinal studies are needed to elucidate the directionality of these relationships and develop strategies to improve dietary intake among OD.

## 1. Introduction

Worldwide obesity rates have tripled in less than five years, with more than 1.9 billion adults categorized as overweight or with obesity in 2016 [1]. Within the United States, high rates of obesity and comorbidities contribute to about USD 150 billion in health care spending [2]. This surge in obesity rates is driven by eating behaviors and poor diet quality, which promote excessive energy intake [3,4]. Olfaction is a critical component of food intake. An extensive body of literature describes the role of olfaction not only in flavor and pleasure perception [5] but also in modifying the preparatory and satiety-related components of dietary intake [6]. Thus, olfactory impairment can diminish flavor perception and reduce enjoyment from eating and drinking [7,8], and may even lead to loss of appetite. Notably, evidence also shows that olfactory dysfunction negatively influences food preference [9], thus shaping dietary intake and quality.

Evidence on the relationship between olfactory dysfunction and dietary intake or diet quality is mixed, though many of these studies included small, relatively homogenous samples. For example, studies show negative [10], positive [11], or no association [7,12] of olfactory dysfunction with fruit and vegetable intake. Similarly, some studies show olfactory dysfunction to be associated with a low intake of sweet, salty, and/or fatty foods [11,13], with others reporting olfactory dysfunction as associated with a higher intake of sugar and fatty foods [7,14,15]. One study focused on diet quality/patterns with olfactory dysfunction. The study found that a reduced capacity to detect odor because of sino-nasal disease was associated with greater consumption of a Western-style diet; however, having a better ability to detect smells demonstrated no association with this dietary pattern [16]. Additionally, a recent publication by Rawal and colleagues documented a low Healthy Eating Index 2015 score (an indicator of diet quality) and high intake of energy-dense diet, total fat, and added sugar in adults with olfactory dysfunction in the 2011–2014 National Health and Nutrition Examination Survey (NHANES) dataset [17]. Roxbury and colleagues also recently reported an association between olfactory dysfunction and a lower intake of micronutrients in U.S. adults [18].

Olfactory functioning is often altered with unhealthy body weight. Adults with obesity are not only more responsive to palatable food odors [19,20], but they also show decreased sensitivity for non-food odors and decreased odor discrimination ability [20] when compared to people with healthy body weight. However, not all individuals with obesity present with significant alterations in olfactory perception. Some reports suggest no association of olfactory perception with body mass index [21]. This discrepancy may be partially explained by the metabolic health of adults with obesity. A recent study showed that the olfactory system of “metabolically” unhealthy individuals with obesity may be more affected compared to “metabolically” healthy individuals. Specifically, these investigators reported that a higher waist-to-hip ratio (an indicator of high visceral fat) was negatively related to odor sensitivity, and this relationship was mediated by insulin resistance independent of BMI status [22]. Thus, the relationship between olfactory dysfunction and dietary intake and quality may be moderated by important metabolic markers, such as body fat percent and waist circumference. Although there is evidence for a potential shift to foods high in sugar, fat, and salt (all hallmarks of ‘Western’ dietary pattern) with olfactory impairments, the mixed findings between olfactory dysfunction and dietary intake and quality warrant further investigations. Specifically, the role of metabolic markers such as body fat percent and waist circumference needs to be examined in a large, more diverse, and representative sample.

Thus, we analyzed data from the 2013–2014 National Health and Nutrition Examination Survey (NHANES), a nationally representative sample of U.S. adults with objective assessments of smell function and dietary intake. We aimed to determine if body fat percent (and waist circumference) moderated the relationship between olfactory function and dietary intake and quality.

## 2. Materials and Methods

### 2.1. Study Design and Sample

The NHANES is a large cross-sectional survey, conducted by the National Center for Health Statistics, designed to monitor the health and nutritional status of non-institutionalized civilians in the U.S. The nationally representative data are collected from 5000 persons of all ages from 15 randomly selected counties/other similar jurisdictions each year and released in two-year increments. The survey oversamples subgroups of the population to improve the precision and reliability and increase the diversity in the estimates of health status. These oversampled subgroups include Hispanics, non-Hispanic Blacks, and Non-Hispanic Asians, Non-Hispanic White and other persons at or below 130% of the poverty level, and Non-Hispanic White and other persons aged 80 years and above. Complete details regarding the NHANES sampling methodology and data collection process are available at the NHANES website (http://www.cdc.gov/nchs/nhanes.htm; accessed on 1 March 2020).

In the 2013–2014 data cycle, NHANES included subjective and objective assessments of smell and taste in adults aged ≥40 years (*n* = 3527) from 30 survey locations across the U.S., in addition to the other traditional health and nutritional measures. Adults were excluded from this examination if they were currently pregnant or breastfeeding. The subjective chemosensory questionnaire was administered during the in-home interview by trained health technicians, using a Computer Assisted Personal Interviewing system. The objective assessments of smell and all collection of dietary data were conducted in mobile examination centers. Approval for data collection was obtained from the NCHS Research Institutional/Ethics Review Board (IRB/ERB) (protocol #2011–17) and all adult study participants provided written consent.

### 2.2. Measures

#### 2.2.1. Olfactory Function Assessment

We included an objective assessment of olfactory function in our analysis. Trained health technicians administered the NHANES eight-item Pocket Smell Test (PST™, Sensonics, Inc., Haddon Heights, NJ, USA). The PST is based on the 40-item University of Pennsylvania Smell Identification Test (UPSIT) [23] and includes four food-related odorants (chocolate, strawberry, grape, and onion), two warning odorants (smoke and natural gas), and two common household odorants (leather and soap). Odorants were microencapsulated and positioned on scent strips on each test card [24,25]. Trained health technicians presented all odors individually by scratching the test strip using a plastic stylus in a fixed order during visits to mobile examination centers. Participants were then asked to smell and identify each odor from a list of four possible responses. To reduce criterion bias, participants were required to choose an answer (forced choice task) even if no odor was perceived. Participants were scored based on the number of correctly identified odors (total score on a scale of 0 to 8). Smell impairment was defined as incorrect identification of three or more odors (resulting total score of 0–5) [24,25], which we labeled as the ‘Olfactory Dysfunction’ (OD) group. Normosmics (6–8 odors correctly identified) were labeled as ‘Normal Olfaction’ (NO).

#### 2.2.2. Dietary Intake and Healthy Eating Index (HEI Calculation)

Dietary intake was collected on two days, with one of the days collected in person by trained interviewers using the automated multiple-pass method of 24 h recall. The second day was completed 3–10 days later over the phone for a slightly smaller group of participants. The complex sampling strategy used for collecting dietary data allows for data from one day of 24 h recall to represent the mean population’s usual intake. The present study includes only data collected during the in-person interview for maximum accuracy, data consistency, and to maximize sample size.

Using this dietary intake data, the NHANES provides calculations for total calories, carbohydrates, protein, fat, fiber, and sodium intake, from which we calculated the percent of calories consumed from carbohydrates, protein, total fat, saturated fatty acids, monounsaturated fatty acids, and polyunsaturated fatty acids. We also constructed a Healthy Eating Index (HEI-2010) score by summing twelve components that reflect the level of adherence to the 2010 U.S. Dietary Guidelines for Americans. Nine of these components are adequacy items (total fruit, whole fruit, total vegetables, green and beans (dark green vegetables and legumes), whole grains, dairy, total protein foods, seafood and plant proteins, and fatty acids), and three components are moderation items (refined grains, sodium, and empty calories from solid fat, alcohol, and added sugars) [26]. The total score ranges from 0 (lowest) to 100 (highest), with higher scores indicating higher diet quality, which reflects greater compliance with the 2010 Dietary Guidelines for Americans. The HEI-2010 component scores are based on the cup or ounce equivalent per 1000 kcal [26]. Component scores are as follows: total fruit (0–5), whole fruit (0–5), total vegetables (0–5), greens and beans (0–5), whole grains (0–10), dairy (0–10), total protein foods (0–5), seafood and plant proteins (0–5), fatty acids (0–10), refined grains (0–10), sodium (0–10), and empty calories (0–20).

#### 2.2.3. Anthropometric and Body Composition Measurement

Body fat percent and waist circumference were measured using standardized protocols by NHANES-trained health technicians at Mobile Examination Centers. To measure body composition, dual-energy X-ray absorptiometry (DXA) whole-body scans were conducted using Hologic Discovery model A densitometers (Hologic, Inc.; Bedford, MA, USA). The scan images were analyzed using APEX v4.0 software (Hologic, Inc.; Bedford, MA, USA) with the NHANES body composition analysis option, which added 5% of lean mass to the fat mass to correct the underestimation of fat mass by this particular densitometer [27]. From this analysis, total fat mass, total lean mass, and bone mass content were derived. Participants were excluded from DXA measurement if pregnant, had amputations other than fingers and toes, had a self-reported history of radiographic contrast material use in the past seven days or participation in nuclear medicine studies in the past three days, weighed over 300 pounds, or had a height over 6′5″.

#### 2.2.4. Covariates

We considered several covariates for these analyses. Participants reported sociodemographic information including age, sex, educational attainment, marital status, race/ethnicity, family income, smoking status, and alcohol intake. Race/ethnicity was categorized as non-Hispanic white, non-Hispanic Black, Mexican American, non-Hispanic Asian, or Other. Family income-to-poverty ratio (IPR) is the ratio of family income to the federal poverty threshold, which was categorized into quartiles: ≤1.1; 1.1–2.2; 2.2–4.3; and >4.3. Educational attainment was categorized as less than high school, high school diploma, some college, or college graduate or more. Employment status was categorized as employed, retired, disabled/unable to work, or other. Adults also self-reported current and lifetime cigarette and alcohol use, which were each categorized as never, current, or past use. Following previous studies using NHANES data, we also considered several other confounders, including whether or not participants reported: (1) smell problems in the past 12 months; (2) a change in the ability to smell since age 25 years; (3) ever having phantom odors; (4) nasal congestion in the past 12 months; (5) ever having their tonsils removed; (6) persistent dry mouth in the past 12 months; or (7) a broken nose, serious injury to the face, skull, or head, or a loss of consciousness.

#### 2.2.5. Data Analysis

Our sample for these analyses included participants with complete data on objective olfactory function, dietary intake, body fat percent, waist circumference, and relevant covariates (*n* = 1415; Supplementary Figure S1). We first described our sample by testing unadjusted differences in participant characteristics by objective olfactory function using ANOVA for continuous variables and Rao–Scott chi-square tests for categorical variables. Similarly, we tested unadjusted differences in dietary intake and quality by objective olfactory function using ANOVA for continuous variables and Rao–Scott chi-square tests for categorical variables. Next, we tested multivariate linear regression models with an interaction term between the objective olfactory function categorical variable and the continuous body fat percent variable for each specific nutrient or diet quality score (or component score) as the outcome. For models with interaction terms, we stratified the models by olfactory function to obtain point estimates of the relationship between body fat percent and the specific nutrient or diet quality score (or component score). We repeated these steps for multivariate models with an interaction between the objective olfactory function categorical variable and the continuous waist circumference variable. All regression models were adjusted for age, sex, IPR, educational attainment, race/ethnicity, and smoking status. All analyses accounted for NHANES complex survey designs and sampling weights using SAS v9.4 (SAS Institute, Cary, NC, USA). Interaction terms have low power and downward bias in non-experimental research. Thus, in accordance with previous research [28,29], including survey research, we set significance at *p* < 0.10 when testing interaction terms. For other comparisons, significance was set at *p* < 0.05.

## 3. Results

### 3.1. Sample Characteristics

**Figure 1:**
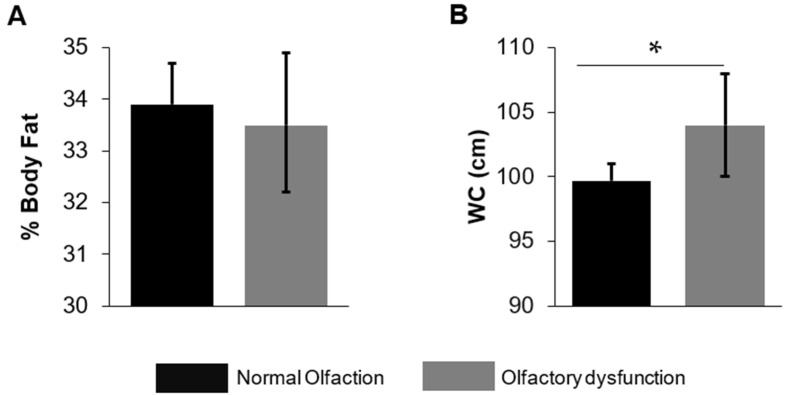
(**A**) Comparison of body fat percent (%) measured using dual-energy X-ray absorptiometry between the two olfactory groups. (**B**) Comparison of waist circumference measured using a measuring tape between the two olfactory groups. * *p* < 0.05 and error bars represent 95% CI.

Of the total analytical sample, 9.5% were categorized with OD based on the objective olfactory assessment. Table 1 describes the socioeconomic, lifestyle, and health characteristics of adults >40 years by objective olfactory function. Figure 1 shows the differences in percent body fat and waist circumference by objective olfactory function. Adults with OD were more likely to be older (OD mean age 50.9 years (95% CI: 49.6, 52.2); NO mean age 49.3 years (48.8, 49.9), male, had less than high school educational attainment, were more likely to be disabled or unable to work, were more likely to be past drinkers, and more likely to have greater waist circumference. Adults with OD were also less likely to be divorced/widowed/separated and less likely to be non-Hispanic white. Adults with OD were more likely to report having smell problems in last 12 months. Although only marginally significant, adults with OD are also more likely to have the lowest IPR.

### 3.2. Dietary Intake and Quality by Objective Olfactory Function

In unadjusted comparisons, we did not observe any statistically significant differences in dietary intake or diet quality between objectively determined NO and OD adults (Table 2).

### 3.3. Moderating Effect of Body Fat or Waist Circumference on the Relationship between Olfactory Function and Dietary Intake

**Figure 2:**
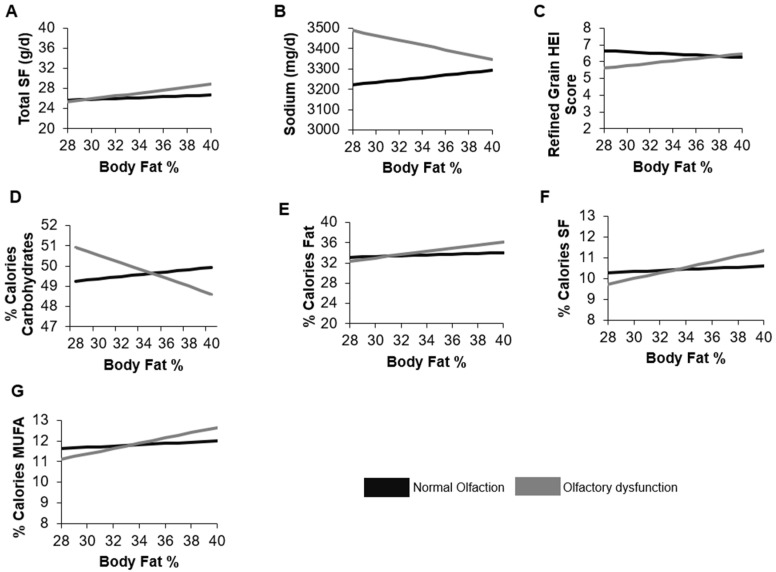
Interaction of body fat percent and olfactory function on: (**A**) total saturated fat, (**B**) sodium, (**C**) refined grain score for the Healthy Eating Index, (**D**) percent of calories consumed as carbohydrates, (**E**) percent of calories consumed as fat, (**F**) percent of calories consumed as saturated fat, (**G**) percent of calories consumed as monounsaturated fatty acid, in adults ≥ 40 years in NHANES 2013–2014. The refined grain score for the Healthy Eating Index ranges from 1 to 10 with higher scores indicating lower (healthier) intake of refined grains. Models adjusted for age, sex, IPR, educational attainment, race/ethnicity, and smoking status. SF: Saturated fat; HEI: Health Eating Index; MUFA: Monounsaturated Fatty Acid.

**Figure 3:**
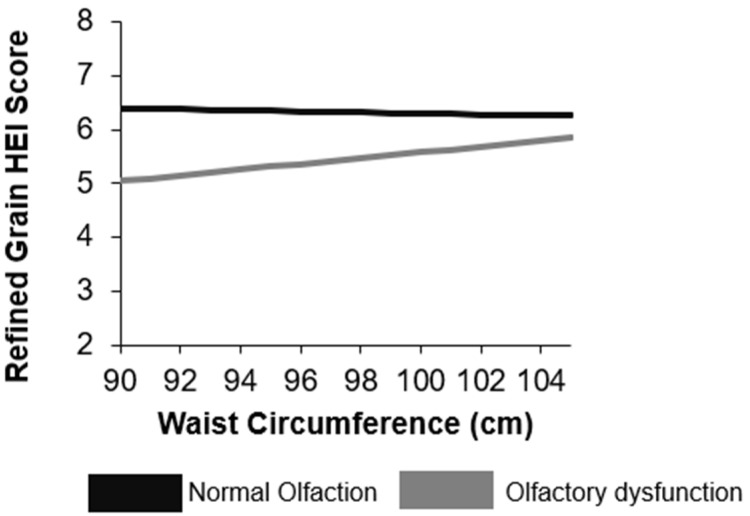
Interaction of waist circumference and olfactory function on Healthy Eating Index score in adults ≥ 40 years in NHANES 2013–2014. The refined grain score for the Healthy Eating Index ranges from 1 to 10 with higher scores indicating lower (healthier) intake of refined grains. Models adjusted for age, sex, IPR, educational attainment, race/ethnicity, and smoking status. HEI: Health Eating Index.

In our tests for the potential effect modification of body fat percent on the relationship between olfactory function and dietary intake and quality, interactions were significant when modeling saturated fat (*p* = 0.06), percent of total calories from fat (*p* = 0.07), percent of total calories from saturated fat (*p* = 0.02), percent of total calories from monounsaturated fat (*p* = 0.08), percent of total calories from carbohydrates (*p* = 0.09), sodium (*p* = 0.08), and HEI refined grain score (*p* = 0.04; Supplementary Table S1). For every one-unit increase in total body fat percent, saturated fat intake was 0.2 ± 0.1 g higher among OD, compared to NO adults (Figure 2). Similarly, each one-unit increase in total body fat was associated with higher percent intake of total calories from total fat (0.2 ± 0.1), saturated fat (0.1 ± 0.04), and monounsaturated fat (0.1 ± 0.1) among OD, compared to NO adults. Every one-unit increase in total body fat percent was also associated with lower intake of percent of total calories from carbohydrates (−0.1 ± 0.1) among OD, compared to NO adults (Figure 2; Supplementary Table S1). Notably, an increase in body fat percent was associated with lower sodium intake (−17.8 ± 9.6 mg) and with a higher (healthier) HEI refined grain component score (0.1 ± 0.1) among OD, compared to NO adults. When testing the interaction of olfactory function and waist circumference on dietary intake and quality, the interaction was only significant for the HEI refined grain component score yet in the opposite direction than expected (Figure 3; Supplementary Table S2). For each one-unit increase in waist circumference, the HEI refined grain component score was 0.01 ± 0.02 points higher among OD, compared to NO adults.

## 4. Discussion

The present study investigated the moderating role of body fat percent and waist circumference, both markers of metabolic health, on the association between olfactory dysfunction and dietary intake and quality, using a large representative sample in the U.S. Our results showed that, with higher body fat, adults with OD differ from adults with NO in their intake of specific dietary components. Specifically, after controlling for covariates, higher body fat percent was associated with higher intake of saturated fat and percent of calories from total fat, saturated fat, and monounsaturated fat, and with lower intake of percent of calories from carbohydrates, among adults with OD compared to adults with NO. Surprisingly, increasing levels of body fat were associated with higher (healthier) HEI refined grain scores and lower sodium among adults with OD, compared to adults with NO. A similar pattern was observed for waist circumference and HEI refined grain scores among adults with OD compared to adults with NO. Together, these results demonstrate that metabolic health likely plays a notable role in shaping dietary intake and quality among adults with OD.

Our study adds to the growing body of literature demonstrating that a normal functioning olfactory system may be critical to consuming a higher-quality diet. The smell is the most important determinant of the flavor of food [30]. Therefore, a reduced ability to smell may decrease pleasure derived from eating. A large survey conducted on patients with chemosensory deficits supports this speculation by showing that participants with chemosensory disorders report pleasure and not nutrition to be critical to their eating [8]. Notably, more than half of the study sample with reduced smell perception reported changing their eating habits, with many reporting higher intake of sweet, salty, bitter, and fatty foods. Similar findings were noted by Zang et al., where patients with olfactory dysfunction rated orthonasal food odors to be less pleasant, less intense, less familiar, and less appetizing [13].

Previous research has demonstrated that metabolic health shapes olfaction. The literature suggests that excess body weight accompanied by abnormal metabolic health parameters (e.g., body fat percent, waist-to-hip ratio, insulin resistance, and leptin) is related to a reduction in olfactory bulb volume [31]. This decline in olfactory bulb volume is associated with lower olfactory performance [32,33]. Data also show that the production of pro-inflammatory factors by adipose tissue in adults with obesity impairs olfactory receptors, thus resulting in chemosensory dysfunction. Excess adipose tissue causes a surge in blood levels of IL-6 and C-reactive protein and enhances the expression of pro-inflammatory cytokines and adipokines [34,35]. Moreover, experimentally enhanced expression of these inflammatory markers has been shown to reduce olfactory sensory neurons in mice fed a high-fat diet [36]. In this context, we examined how a greater abnormality in body weight-related metabolic markers may impact adults with olfactory dysfunction by influencing their dietary intake and quality. Indeed, we found that among adults with OD, higher body fat percentage was associated with a greater intake of percent calories consumed as total fat, saturated fat, and monounsaturated fat. Surprisingly, sodium, percent of calories from carbohydrates, and refined grains were lower among OD participants with increasing levels of percent body fat. One potential explanation of these mixed findings for dietary intake and quality among adults with OD is interindividual variability in eating behaviors with the loss of smell. For example, some patients with olfactory loss have reported changes in their food-related social activities, such as decreased frequency of eating at restaurants and lower interest in meeting people for dinner [11]. Because meals away from home tend to be higher in energy density, dietary fat, etc., but lower in fruits, vegetables, whole grains, etc. [37], OD adults with reduced frequency of dining out may also have a lower intake of sodium and carbohydrates. This, however, is speculation as we did not include the frequency of eating away from home in our analyses. Furthermore, in a previous study only 30% of participants with hyposmia self-reported an increase in salt usage to compensate for diminished flavor perception, while 70% of hyposmic participants did not report any change in salt usage [38]. This further supports the variability in compensatory behavior and, thus, may help explain the lower sodium intake among OD participants in our sample. While we did not assess taste perception, high circulating leptin with high body fat increases the threshold for sweet taste [39]. Adults in our sample with high body fat may have had high leptin and thus were consuming sugary foods with high-fat content instead of items with high salt and carbohydrate content. Likewise, adults in our sample with higher body fat percent and WC may have been attempting to make dietary changes to lose weight by following a low carbohydrate diet. A low carbohydrate dietary regimen is generally accompanied by a high-fat intake and is an effective strategy for weight loss [40].

Our finding that adults with OD did not report higher total calorie intake compared to the group with NO is supported by other studies [14,17,41]. Our report of a greater intake of calories from total fat and saturated fat among OD adults with higher levels of body fat aligns with a recent comparison of energy density between OD and NO in the US NHANES dataset [17] but does not support the reports from the Korean NHANES dataset [14]. Among adults in the Korean NHANES, especially young and middle-aged females, olfactory dysfunction was related to reduced total fat intake, which was believed to be related to lower intake of fat n-6 PUFAs, margarine, nuts, and fish [14], dietary components considered protective towards olfactory impairment [42]. However, this assumption could not be formally tested due to the cross-sectional nature of the data in the Korean NHANES dataset. Overall, our findings show an unhealthy shift in the composition of calories indicating a move towards a Western type of dietary pattern. While our analyses were performed on a cross-sectional dataset, a longitudinal analysis over five years demonstrated that women with olfactory impairments were less likely to adhere to a healthy diet [43], suggesting a potential long-term impact of olfactory function on dietary intake.

Our study has several strengths. First, the data on olfactory dysfunction were collected by rigorously trained technicians who followed a standardized protocol. Most importantly, a validated objective measure was used to determine olfactory dysfunction in all participants. This is critical because a recent meta-analysis showed that reports of olfactory dysfunction in the general population depend on the testing methods, such that the percent of people reporting olfactory dysfunction is greater using objective olfactory assessment compared to subjective measures [44]. Further, our analysis was performed on a large national population sample in the U.S. We also controlled for a variety of covariates that can shape olfactory function, metabolic markers, and dietary intake. Specifically, as noted in previous studies, sex and age can significantly influence olfactory function [45,46,47]. Thus, controlling for these and other potential confounders is critical for our analysis; although, residual confounding is still possible.

Despite these strengths, there are some limitations to consider. Our study was cross-sectional in design, so reverse causality is possible. Furthermore, despite NHANES being a large dataset, the number of individuals with OD was small compared to the participants with NO. Additionally, the 24 h diet recall data used to determine dietary components and diet quality were self-reported in nature and only the first of the two-day recall was used to maximize sample size. Substantial evidence suggests that traditional methods of self-reported dietary data collection are prone to reporting errors and appear to underestimate energy and nutrient intake [48,49]. Thus, our findings should be interpreted with this limitation in mind. Moreover, the HEI scores calculated to determine diet quality are specific to dietary standards recommended in the U.S. Thus, the results cannot be generalized to populations outside the U.S., and some other important dietary components relevant to more diverse populations may have been missing. Further, we did not include taste evaluation in our analysis. Interactions between body fat and olfactory function are complex. Internal metabolic signals such as fluctuation in glucose and other hormonal and metabolic changes in individuals with obesity can influence sensory perception. Adipokines and cytokines secreted from visceral fat may cause alterations in olfactory function [50]. The level of fat accumulation can also influence leptin resistance, modulating olfactory sensitivity. Extensive evidence demonstrates the influence of hunger and satiety states on olfactory sensitivity [51]. All these factors need to be kept in mind when interpreting our findings.

Altogether, after controlling for confounders in a nationally representative dataset from the U.S., we showed that the relationship between olfactory dysfunction and diet quality was moderated by the body fat percent. Our findings provide a basis for future studies with a larger number of participants with olfactory dysfunction and more objective markers of metabolic health. Further, longitudinal and experimental study designs will be critical to assess the directionality of the relationship between olfactory dysfunction and dietary changes and develop potential strategies to improve dietary intake. Future studies should also consider how olfactory dysfunction and higher levels of body fat may be contributing to inequities in dietary intake and metabolic health. The simultaneous increase in screening and treatment for both olfactory dysfunction and markers of metabolic health may be a pathway to improving dietary intake and quality and reducing health inequities.

## Figures and Tables

**Table 1:** Weighted characteristics by objective olfactory function among U.S. adults > 40 years in NHANES 2013–2014.

Objective Olfactory Function ^1^	Normal Olfaction (6–8 Odors Correct)	Olfactory Dysfunction (<6 Odors Correct)	*p*-Value
Characteristic	*n* = 1280	*n* = 135	
Age, years	49.3 (48.8, 49.9)	50.9 (49.6, 52.2)	0.01
Female	50.7 (46.8, 54.6)	37.3 (30.5, 44.2)	<0.0001
Educational attainment			<0.0001
Less than high school	13.5 (8.8, 18.3)	31.2 (21.2, 41.3)	
High school diploma	22.4 (17.4, 27.4)	16.6 (9.0, 24.3)	
Some college	30.4 (24.9, 36.0)	21.9 (16.2, 27.6)	
College graduate or more	33.6 (27.6, 39.6)	30.2 (18.5, 41.8)	
Marital status			0.04
Married/Partner	70.5 (66.2, 74.8)	74.0 (61.5, 86.5)	
Divorced/Widowed/Separated	23.4 (18.1, 25.1)	11.0 (3.9, 18.1)	
Never married	7.9 (5.6, 10.1)	15.0 (6.4, 23.7)	
Income-to-poverty ratio (IPR) ^2^			0.06
IPR ≤ 1.1	13.8 (7.3, 20.3)	21.6 (11.2, 31.9)	
1.1 < IPR ≥ 2.2	17.1 (13.7, 20.5)	18.8 (8.7, 29.0)	
2.2 < IPR ≥ 4.3	23.8 (18.9, 28.8)	28.5 (17.9, 39.1)	
IPR > 4.3	39.4 (31.2, 47.5)	20.3 (4.5, 36.2)	
Missing	5.9 (3.1, 8.7)	10.8 (1.6, 20.0)	
Employment status			0.51
Employed	75.4 (67.2, 83.5)	68.6 (53.3, 83.9)	
Retired	1.7 (0.7, 2.7)	2.0 (0, 4.8)	
Disabled/unable to work	10.8 (5.4, 16.2)	16.7 (8.7, 24.7)	
Other	12.1 (9.3, 14.9)	12.8 (2.9, 22.6)	
Race/Ethnicity			0.03
Mexican American	8.2 (4.4, 12.0)	10.1 (1.7, 18.5)	
Non-Hispanic White	66.8 (58.4, 75.1)	53.0 (39.7, 66.3)	
Non-Hispanic Black	10.8 (7.5, 14.0)	17.4 (9.5, 25.3)	
Non-Hispanic Asian	5.5 (3.3, 7.7)	10.4 (3.3, 17.5)	
Other	8.8 (5.2, 12.4)	9.1 (2.5, 15.7)	
Smoking			0.61
Never	56.7 (50.4, 63.1)	54.6 (47.1, 62.2)	
Current	21.8 (16.6, 27.0)	26.6 (16.0, 37.2)	
Past	21.4 (18.9, 24.0)	18.8 (7.8, 29.7)	
Alcohol intake			0.02
Never	80.3 (76.8, 83.7)	69.0 (58.2, 79.8)	
Current	8.0 (5.8, 10.2)	9.8 (3.9, 15.6)	
Past	8.2 (5.0, 11.6)	13.2 (636, 20.2)	
Missing	3.4 (2.2, 4.7)	8.0 (1.9, 14.1)	
Had problems with smell in past 12 months	6.6 (4.2, 9.0)	18.2 (7.5, 28.9)	0.002
Had change in ability to smell since age 25 years	7.6 (4.9, 10.4)	5.6 (2.0, 9.1)	0.41
Have phantom odors	8.1 (5.5, 10.7)	8.7 (0, 17.6)	0.87
Frequent nasal congestion in past 12 months	30.1 (26.3, 34.0)	33.2 (22.8, 43.6)	0.53
Ever had tonsils removed	24.9 (20.6, 29.3)	20.1 (6.1, 34.0)	0.46
Had persistent dry mouth in past 12 months	10.1 (7.7, 12.5)	11.9 (4.6, 19.2)	0.61
Injury to nose, face, head, or skull, or loss of consciousness	25.8 (18.9, 32.6)	23.1 (13.7, 32.6)	0.57
Waist circumference, cm	99.7 (98.3, 101)	104 (100, 108)	0.03
Body fat percent, %	33.9 (33.1, 34.7)	33.5 (32.1, 34.8)	0.53

Data are shown as a mean (95% CI) for age, waist circumference, and body fat percent. All other values are shown as % (95% CI). ^1^ Objective olfactory function was assessed with the eight-item Pocket Smell Test. ^2^ Income-to-Poverty Ratio is the ratio of family income to federal poverty guidelines.

**Table 2:** Mean (95% CI) dietary intake by objective olfactory function among U.S. adults > 40 years in NHANES 2013–2014.

Objective Olfactory Function ^1^	Normal Olfaction (6–8 Odors Correct)	Olfactory Dysfunction (<6 Odors Correct)	*p*-Value
Nutrient or Dietary Quality	*n* = 1280	*n* = 135	
Total energy intake, kcal	2167 (2099, 2235)	2184 (1908, 2462)	0.89
Total fat (g/d)	83.6 (80.7, 86.5)	87.1 (67.4, 107)	0.70
Saturated fat	27.0 (26.0, 28.0)	27.4 (22.5, 32.4)	0.84
Monounsaturated fat	29.3 (28.3, 30.3)	30.0 (22.6, 37.4)	0.83
Polyunsaturated fat	19.3 (18.3, 20.4)	21.8 (14.9, 28.6)	0.46
Percent of total calories from fat	34.2 (33.6, 34.8)	34.4 (31.5, 37.4)	0.85
Percent of total calories from saturated fat	10.9 (10.7, 11.2)	10.9 (10.2, 11.6)	0.95
Percent of total calories from monounsaturated fat	12.0 (11.7, 12.2)	11.9 (10.5, 13.2)	0.91
Percent of total calories from polyunsaturated fat	8.0 (7.7, 8.3)	8.3 (7.0, 9.6)	0.71
Protein (g/d)	85.2 (82.7, 87.7)	84.3 (74.4, 94.2)	0.86
Percent of total calories from protein	16.1 (15.4, 16.7)	16.7 (14.9, 18.4)	0.47
Total carbohydrates (g/d)	254 (245, 264)	262 (231, 293)	0.63
Percent of total calories from carbohydrates	47.5 (46.5, 48.4)	48.4 (46.2, 50.6)	0.43
Dietary fiber (g/d)	17.3 (16.5, 18.1)	18.0 (14.3, 21.7)	0.68
Sodium (mg/d)	3585 (3484, 3687)	3763 (3349, 4177)	0.40
Total HEI score ^1^	51.5 (49.5, 53.5)	49.0 (43.0, 55.1)	0.39
Total fruit score	1.9 (1.7, 2.2)	1.8 (1.4, 2.2)	0.55
Whole fruit score	2.1 (1.8, 2.4)	1.8 (1.1, 2.4)	0.36
Total vegetable score	3.1 (2.9, 3.3)	2.9 (2.5, 3.2)	0.11
Greens and beans score	1.3 (1.1, 1.6)	1.4 (0.9, 1.8)	0.94
Whole grain score	2.8 (2.5, 3.0)	2.0 (0.8, 3.2)	0.26
Dairy score	4.8 (4.6, 5.1)	5.3 (4.2, 6.3)	0.41
Total protein score	4.3 (4.2, 4.3)	4.2 (3.9, 4.6)	0.93
Seafood and plant protein score	2.2 (1.9, 2.5)	1.8 (1.3, 2.3)	0.18
Fatty acid score	5.2 (5.0, 5.5)	5.1 (4.1, 6.0)	0.71
Refined grain score	6.2 (6.0, 6.5)	5.6 (4.5, 6.6)	0.16
Sodium score	4.4 (4.1, 4.7)	3.8 (2.9, 4.7)	0.20
Empty calorie score ^2^	13.2 (12.5, 14.0)	13.5 (12.2, 14.7)	0.73

Data are shown as mean (95% CI). ^1^ The Healthy Eating Index (HEI) score includes nine dietary components based on the 2010 U.S. Dietary Guidelines for Americans. Higher scores indicate higher dietary quality (0–100). ^2^ The empty calorie score component of the HEI captures three moderation areas of dietary intake: solid fats, alcohols, and added sugars.

## Data Availability

Data described in the manuscript, code book, and analytic code will be made available upon request pending application and approval.

## Supplementary Materials

The following supporting information can be downloaded at: https://www.mdpi.com/article/10.3390/nu14153178/s1, Figure S1: Participant Inclusion Flow Chart; Table S1: Estimates (SE) for the association between the interaction of olfactory function status and body fat percent with dietary intake among U.S. adults >40 years in NHANES 2013–2014; Table S2: Estimates (SE) for the association between the interaction of olfactory function status and waist circumference with dietary intake among U.S. adults >40 years in NHANES 2013–2014.

## Abbreviations

HEI	Healthy Eating Index
NHANES	National Health and Nutrition Examination Survey
NCHS	National Center for Health Statistics
IPR	Family income-to-poverty ratio
DXA	Dual-Energy X-ray absorptiometry
UPSIT	University of Pennsylvania Smell Identification Test
PST™	Pocket Smell Test
