# Supplementary material for: Body Fat Moderates the Association of Olfactory Dysfunction with Dietary Intake in U.S. Middle-Aged and Older Adults: A Cross-Sectional Analysis of NHANES 2013–2014
Source: Nutrients. 2022 Aug 2;14(15):3178. doi: 10.3390/nu14153178 (PMC9370378; doi:10.3390/nu14153178)
Supplement: Supplementary file 1 [file nutrients-14-03178-s001.zip › nutrients-1805154-supplementary.pdf]

Online Supplementary Material

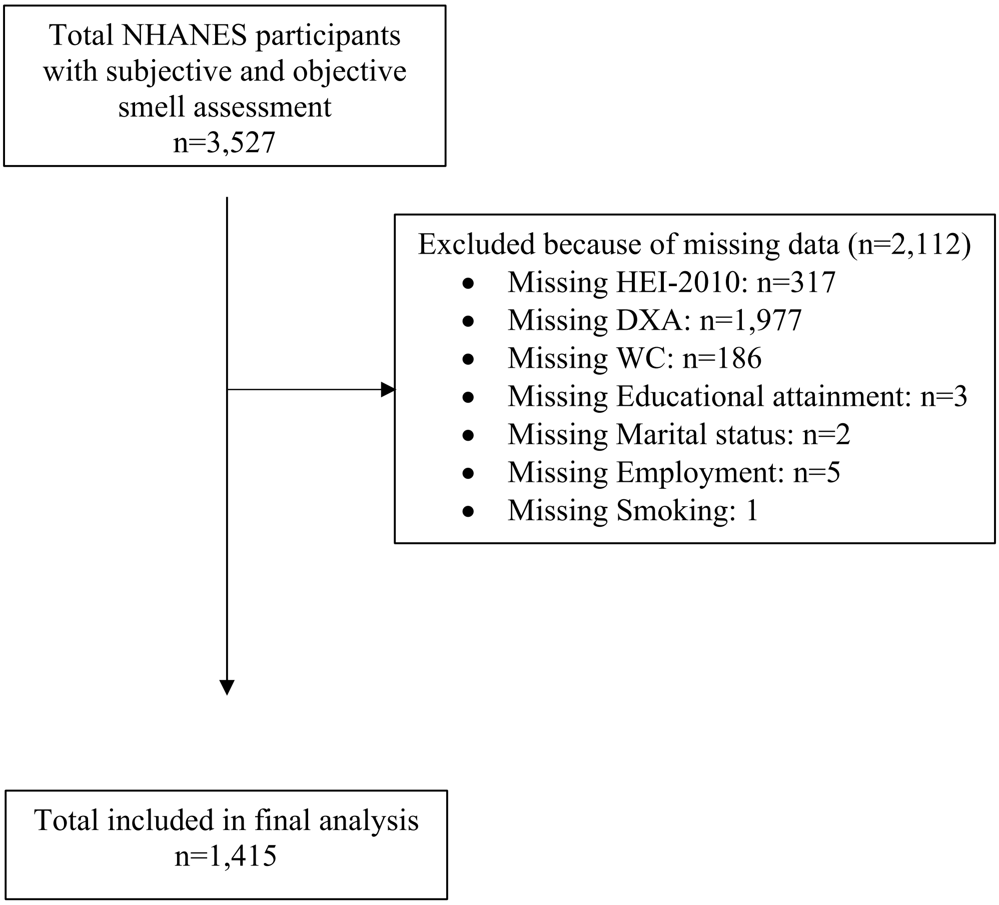

Supplementary Figure S1. Participant Inclusion Flow Chart. WC: Waist Circumference, HEI: Healthy Eating Index, DXA: Dual-Energy X-ray absorptiometry.

Supplementary Table S1. Estimates (SE) for the association between the interaction of olfactory function status and body fat percent with dietary intake among U.S. adults >40y in NHANES 2013-2014.

Nutrient or dietary quality	Estimate (SE)	P-value
Total energy intake, kcal	2.2 (16.2)	0.90
Total fat (g/d)	0.5 (0.3)	0.16
Saturated fat	0.2 (0.1)	0.06
Monounsaturated fat	0.2 (0.2)	0.13
Polyunsaturated fat	0.03 (0.1)	0.81
Percent of total calories from fat	0.2 (0.1)	0.07
Percent of total calories from saturated fat	0.1 (0.04)	0.02
Percent of total calories from monounsaturated fat	0.1 (0.1)	0.08
Percent of total calories from polyunsaturated fat	0.02 (0.04)	0.64
Protein (g/d)	-0.3 (0.4)	0.48
Percent of total calories from protein	0.03 (0.1)	0.74
Total carbohydrates (g/d)	-0.8 (0.8)	0.30
Percent of total calories from carbohydrates	-0.2 (0.1)	0.09
Dietary fiber (g/d)	-0.2 (0.2)	0.25
Sodium (mg/d)	-17.8 (9.6)	0.08
Total HEI score[1]	0.1 (0.1)	0.68
Total fruit score	-0.01 (0.03)	0.78
Whole fruit score	-0.03 (0.03)	0.36
Total vegetable score	0.003 (0.02)	0.86
Greens and beans score	-0.02 (0.02)	0.26
Whole grain score	-0.02 (0.04)	0.66
Dairy score	0.04 (0.03)	0.19
Total protein score	0.01 (0.01)	0.42
Seafood and plant protein score	0.03 (0.03)	0.37
Fatty acid score	-0.02 (0.03)	0.54
Refined grain score	0.1 (0.1)	0.04
Sodium score	0.02 (0.03)	0.51
Empty calorie score[2]	-0.06 (0.1)	0.33

[1]The Healthy Eating Index (HEI) score includes nine dietary components based on the 2010 U.S. Dietary Guidelines for Americans. Higher scores indicate higher dietary quality (0-100).
[2]The empty calorie score component of the HEI captures three moderation areas of dietary intake: solid fats, alcohols, and added sugars. Models adjusted for age, sex, IPR, educational attainment, race/ethnicity, and smoking status.

Supplementary Table S2. Estimates (SE) for the association between the interaction of olfactory function status and waist circumference with dietary intake among U.S. adults >40y in NHANES 2013-2014.

Nutrient or dietary quality	Estimate (SE)	P-value
Total energy intake, kcal	3.1 (7.5)	0.69
Total fat (g/d)	0.3 (0.3)	0.319
Saturated fat	0.1 (0.2)	0.22
Monounsaturated fat	0.1 (0.1)	0.49
Polyunsaturated fat	0.1 (0.1)	0.36
Percent of total calories from fat	0.1 (0.1)	0.22
Percent of total calories from saturated fat	0.04 (0.02)	0.11
Percent of total calories from monounsaturated fat	0.02 (0.03)	0.55
Percent of total calories from polyunsaturated fat	0.03 (0.04)	0.38
Protein (g/d)	-0.1 (0.2)	0.54
Percent of total calories from protein	0.01 (0.01)	0.90
Total carbohydrates (g/d)	-0.1 (0.8)	0.89
Percent of total calories from carbohydrates	-0.01 (0.1)	0.51
Dietary fiber (g/d)	-0.1 (0.1)	0.20
Sodium (mg/d)	-5.7 (9.7)	0.57
Total HEI score[1]	0.1 (0.1)	0.45
Total fruit score	-0.01 (0.1)	0.47
Whole fruit score	-0.01 (0.01)	0.29
Total vegetable score	0.01 (0.01)	0.73
Greens and beans score	-0.003 (0.01)	0.77
Whole grain score	0.02 (0.02)	0.26
Dairy score	0.03 (0.03)	0.28
Total protein score	0.0003 (0.01)	0.97
Seafood and plant protein score	0.01 (0.02)	0.69
Fatty acid score	-0.01 (0.03)	0.64
Refined grain score	0.01 (0.02)	0.01
Sodium score	-0.01 (0.03)	0.76
Empty calorie score[2]	-0.03 (0.1)	0.50

[1]The Healthy Eating Index (HEI) score includes nine dietary components based on the 2010 U.S. Dietary Guidelines for Americans. Higher scores indicate higher dietary quality (0-100).
[2]The empty calorie score component of the HEI captures three moderation areas of dietary intake: solid fats, alcohols, and added sugars. Models adjusted for age, sex, IPR, educational attainment, race/ethnicity, and smoking status.
